# Cactus pear: a natural product in cancer chemoprevention

**DOI:** 10.1186/1475-2891-4-25

**Published:** 2005-09-08

**Authors:** Da-ming Zou, Molly Brewer, Francisco Garcia, Jean M Feugang, Jian Wang, Roungyu Zang, Huaguang Liu, Changping Zou

**Affiliations:** 1 Department of Obstetrics and Gynecology, Arizona Health Sciences Center, University of Arizona, Tucson, Arizona 85724, USA; 2 Division of Gynecologic Oncology, Arizona Cancer Center, Tucson, Arizona 85724, USA; 3 Department of Gynecologic Oncology, Fudan Univeristy, Shanghai, 200032, China; 4 Guangxi Medical University, Guangxi, 532021, China

## Abstract

**Background:**

Cancer chemoprevention is a new approach in cancer prevention, in which chemical agents are used to prevent cancer in normal and/or high-risk populations. Although chemoprevention has shown promise in some epithelial cancers, currently available preventive agents are limited and the agents are costly, generally with side effects. Natural products, such as grape seed, green tea, and certain herbs have demonstrated anti-cancer effects. To find a natural product that can be used in chemoprevention of cancer, we tested Arizona cactus fruit solution, the aqueous extracts of cactus pear, for its anti-cancer effects in cultured cells and in an animal model.

**Method:**

Aqueous extracts of cactus pear were used to treat immortalized ovarian and cervical epithelial cells, as well as ovarian, cervical, and bladder cancer cells. Aqueous extracts of cactus pear were used at six concentrations (0, 0.5, 1, 5, 10 or 25%) to treat cells for 1, 3, or 5 days. Growth inhibition, apoptosis induction, and cell cycle changes were analyzed in the cultured cells; the suppression of tumor growth in nude mice was evaluated and compared with the effect of a synthetic retinoid N-(4-hydroxyphernyl) retinamide (4-HPR), which is currently used as a chemoprevention agent. Immunohistochemistry staining of tissue samples from animal tumors was performed to examine the gene expression.

**Results:**

Cells exposed to cactus pear extracts had a significant increase in apoptosis and growth inhibition in both immortalized epithelial cells and cancer cells in a dose- and time-dependent manner. It also affected cell cycle of cancer cells by increasing G1 and decreasing G2 and S phases. Both 4-HPR and cactus pear extracts significantly suppressed tumor growth in nude mice, increased annexin IV expression, and decreased VEGF expression.

**Conclusion:**

Arizona cactus pear extracts effectively inhibited cell growth in several different immortalized and cancer cell cultures, suppressed tumor growth in nude mice, and modulated expression of tumor-related genes. These effects were comparable with those caused by a synthetic retinoid currently used in chemoprevention trials. The mechanism of the anti-cancer effects of cactus pear extracts needs to be further studied.

## Background

The goal of cancer prevention is to delay or block the processes of initiation and progression from pre-cancerous cells into cancer. Cancer chemoprevention, which targets normal and high risk populations, involves the use of drugs or other chemical agents to inhibit, delay, or reverse cancer development [1,2]. There has been significant success in the study of cancer prevention and chemoprevention in the last 20 years [1,2] and, as a result, the incidences of certain types of cancer have decreased due to prevention techniques and improved screening technology [1,2]. However, the incidence and mortality rates of ovarian cancer have remained essentially unchanged [3], partially because early detection methods (primary prevention) have not been developed and prevention of recurrence (secondary prevention) has not been achieved. Furthermore, only a limited number of potentially useful chemopreventive agent(s) have been tested [2,4-6]. Discovery and development of dietary agents for cancer prevention first began at the National Cancer Institute in 1987 [7]. Although hundreds of agents have been developed in the United States during the past decade, only a few new drugs have actually been approved [7,8]. The development of chemopreventive agents is slow and inefficient. More effective and less toxic agents, including natural products, are needed if we are to reach the goal of cancer prevention, both primary and secondary.

A synthetic retinoid, N-(4-hydroxyphernyl) retinamide (4-HPR), was found to decrease the risk of ovarian cancer in an Italian breast cancer chemoprevention trial [9-11]. Women receiving 4-HPR demonstrated a decreased incidence of ovarian cancer [9,10]; however, after cessation of the treatment, ovarian cancer did develop in the treatment group [10,11]. Other studies have also reported that the response of retinoids was not durable in pre-cancer and cancer treatments for either oral leukoplakia or cervical cancer [13-16]. These reports suggest that long-term administration of agents with lower toxicity will be the most important aspect in chemopreventive agents, especially for normal and high risk populations.

Medical benefits from plant forms have been recognized for centuries. Herbs have been used in Chinese medicine for thousands of years to cure diseases and heal wounds. Recently, it has been found that components in green tea and grape seeds have anticancer effects [17,18]. Also, as a rule, herbs and natural products lack much of the toxicity that is present in synthetic chemicals, thus, enhancing their appeal for long term preventive strategies.

Cactus (*Opuntia*) has been used for many years as a common vegetable and as medicine by the Native Americans and Mexicans [19-22]. Cactus contains a fruit known as cactus pear (*Opuntia ficus-indica*) and the plant is referred to as nopale (pad). Cactus pear contains pectin, carotenes, betalains, ascorbic acid, quercetina and quercetin derivatives all of which have antioxidant activity [21-24]. In Chinese medicine, cactus fruit is considered a weak poison and used as medicine for treatment of inflammation and pain [23,24]. It has also been used as a detoxification agent for snake bite [23,24].

In this study, we tested aqueous extracts of cactus pear for its anti-cancer effects in ovarian, cervix, and bladder cancer cells, and in the nude mice ovarian animal model. These results were compared to the effect of 4-HPR, demonstrated the anti-cancer effect of the cactus pear.

## Methods

### Cell lines

The immortalized ovarian epithelium cells (IOSE), the ovarian cancer cell lines OVCA420, SKOV3; the HPVE6 immortalized cervical epithelium cell line TCL-1; cervical cancer cell lines, HeLa and Me180; and bladder cancer cells UM-UC-6, T24, were all used in this study. Cells were grown in a 1:1 (v/v) mixture of Dulbecco's modified Eagle's medium (DMEM) and Ham's F12 with 10% fetal bovine serum at 37°C in a humidified atmosphere of 95% air and 5% CO_2_.

### Animals

Athymic 4 to 6 weeks old nu/nu BALB/c female mice were purchased from the Animal Production Area at the National Cancer Institute, Frederick Cancer Research Facility (Frederick, MD). The mice were housed in laminar flow cabinets under pathogen-free conditions and maintained at the University of Arizona's Animal Care Facility in the College of Medicine, according to institutional regulations approved by the Animal Welfare Committee as well as current regulations and standards of the Department of Agriculture and the Department of Health and Human Services.

### Cactus product

The cactus pear extract was purified from mature cactus fruit by blending. The cactus pear solution contained both the fruit of the cactus and the seeds, and were centrifuged at 4,000 RPM for 30 min and filtered using a 0.45 μM Nalgene filter (Rochester, NY), then aliquoted to 15 ml and stored at -20°C. We used pure extracts which were diluted in cell culture medium to achieve concentrations of 0, 0.5, 1, 5, 10 and 25% (v/v), before being used in cell culture. The osmolality of the solution was 358 m Osm/kg for 25% solution, 342 m Osm/kg for 10%, and 326 m Osm/kg for 5%. The pH was between 7.26–7.28. Animals were treated with pure cactus pear fruit intraperitoneally (i.p.) at 0.4 ml per day.

### Effects of cactus products on cell proliferation in monolayer cultures

Cells were plated in 96-well plates at a concentration of 10^4 ^cells per well and grown for 24 hours. The cells were then incubated in cactus pear solution at different concentrations for 1, 3, or 5 days. Growth inhibition was determined using the crystal violet method, as described [25]. Briefly, after 5 days of treatment, cells were fixed by 5% glutartaldehyde in phosphate-buffered saline (PBS), rinsed with distilled water, and dried completely. Cells were incubated in a 1:1 (v/v) mixture of 200 mM 3-(cyclohexylamino)-1-propanesulfonic acid (CAPS; pH 9.5) and 0.2% crystal violet at 25°C for 30 min, and then were washed and dried. The fixed and stained cells were solubilized with 10% glacial acetic acid, and absorbance at A590 nm was determined using a plate reader. Growth inhibition was calculated according to the equation: inhibition = (1-Nt/Nc) × 100, where Nt and Nc are the numbers of cells in treated and control cultures, respectively.

All experiments were performed in triplicate and the mean ± standard deviations were calculated. IC50 were also determined at 50% of cell growth rate in each.

### Cell cycle analysis by propidium iodide (PI) staining

Cells were treated with 0, 5 and 25% of cactus pear solution for 2 days, were collected by centrifugation and fixed in 4% paraformaldehyde pH 7.4 at room temperature for 30 min, and washed and incubated in 70% ethanol containing 1% HCl at -20°C for 10 minutes. Cells were then stained with 500 μl of propidium iodide/RNase A solution in the dark for 30 min at room temperature, analyzed by flow cytometry using a FACScan flow cytometer (BD Biosciences, San Jose, CA) with a 15 mW Argon laser used for excitation at 488 nm. Fluorescence was measured at 585 nm. Computer analysis was completed using BD Biosciences Cellquest Pro and ModFit LT by Verity Software data processing to provide information on the percentage of apoptotic cells as well as the proportion of cells in G1, S, and G_2 _phases of the cell cycle.

### Analysis of apoptosis induced by cactus product by terminal deoxynucleotidyl transferase (TdT)-mediated fluorescein-deoxyuridine-triphosphate (dUTP) nick-end labeling (TUNEL) assay [25]

Following incubation with 0, 5, and 25% cactus pear solution for 2 days, cells were fixed in 1% formaldehyde in PBS (pH 7.4) for 15 min at 4°C. The cells were then washed twice with PBS, resuspended in 70% ice-cold ethanol and stored in a -20°C freezer until use. For the assay, cells were first suspended in 1 ml wash buffer containing cacodylic acid, Tris-HCl buffered solution and sodium azide (Phoenix flow cytometry kit, Phoenix Flow Systems, San Diego, CA). Approximately 10^6 ^cells were resuspended in 50 μl staining buffer containing Tris-HCl buffer, TdT, and fluorescein-12-dUTP (Phoenix flow cytometry kit). Cells were incubated at 37°C for 60 min, and then rinsed twice with PBS. Cells were stained with 500 μl of propidium iodide/RNase A solution in the dark for 30 min at room temperature and then analyzed by flow cytometry using a FACScan flow cytometer (Epics Profile, Coulter Corp., Hialeah, FL) with a 15 mW argon laser used for excitation at 488 nm. Fluorescence was measured at excitation 520 nm and 570 nm. The Phoenix flow cytometry kit included suspensions of cells that served as negative and positive controls for apoptosis. Computer analysis of the data provided information on the percentage of apoptotic cells as well as the proportion of cells in the hypodiploid, G1, S, and G_2 _phases of the cell cycle.

### Human tumor xenografts

**Figure 1:**
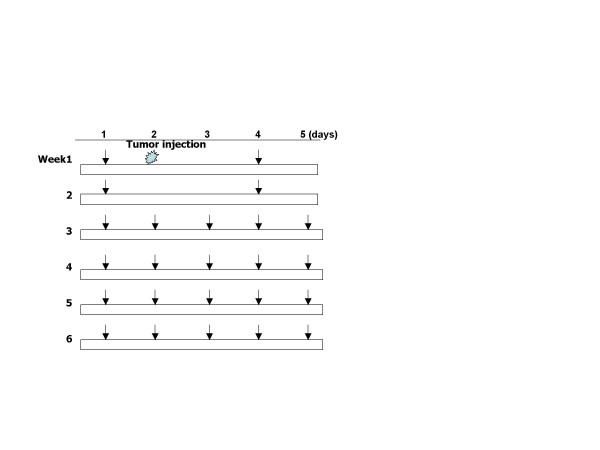
Cactus pear extracts treatment schedule in animal. Numbers of injection times/week were represented by arrow bars. Four groups of animal were examined in the study: SKOV3 alone, SKOV3 + H_2_O, SKOV3 + Cactus extracts, and SKOV3 + 4HPR. 4-HPR concentration was used at 0.43 mg/kg, which equivalent to human 200 mg/kg.

Ovarian cancer cells SKOV3 were grown to sub-confluence and harvested using 0.1% trypsin and 1 mM EDTA. The cells were washed with serum containing medium to quench the trypsin and then with serum-free medium. Cell viability was determined by Trypan blue exclusion and only cultures with more than 90% viability were used for the *in vivo *experiments. The cells were resuspended in medium at 5 × 10^6 ^cells. Cactus pear solution, as well as the chemopreventive agent 4-HPR (0.43 mg i.p twice/week, which equivalent to 200 mg/kg human dose) were injected one day prior to tumor cell injection (day 1) (Fig. 1). Control animals received H_2_O. Tumor cells were injected subcutaneously (day 2). The tumors appeared on day 10–14, and their size was measured twice a week using a caliper. The larger (A) and smaller (B) diameters were used to calculate the tumor volume (V) by using the equation V = 0.4 × A × B^2 ^[27]. The treatment regimen of cactus pear solution was as follows: 0.4 ml of solution injected i.p. twice a week for the first two weeks, then five times a week from the third week to the sixth week (Fig. 1).

### Immunostaining

Paraffin-embedded sections were deparaffinized in xylene, rehydrated through graded alcohols to water, then incubated for 10 min in PBS. The sections were blocked for 30 min with 3% normal horse serum (NHS) diluted in PBS; the sections were then blotted and incubated with p53, annexin IV and VEGF antibodies (Santa Cruz Biotech, Santa Cruz, CA, and Zymed Lab Inc, San Francisco, CA) for 1 hr at room temperature. The endogenous peroxidase was inactivated by incubation for 30 min in 0.015% peroxide in methanol and rehydrated for 10 min in PBS. The slides were incubated with biotinylated horse antibody for 1 hr and washed in PBS, followed by the avidin-biotin-peroxidase complex (ABC, Vector Laboratories, Burlingame, CA). The slides were washed and the peroxidase reaction developed with diaminobenzidine and peroxide, then counterstained with hemotoxylin, mounted in aqua-mount, and evaluated on a light microscopy. Positive and negative antibodies and bladder and ovarian cancer cells were used as controls in each assay.

### Statistical analysis

Student's *t *test was performed to compare two means. One-way ANOVA, followed by the Fisher's Least Square Difference (LSD) test, was used to analyze tumor size in different treatment groups or multiple means. Two-sided P values were determined in all analyses. P < 0.05 is considered as statistically significant.

## Results

### Growth inhibitory effect of cactus pear solution on human ovarian cell lines

**Figure 2:**
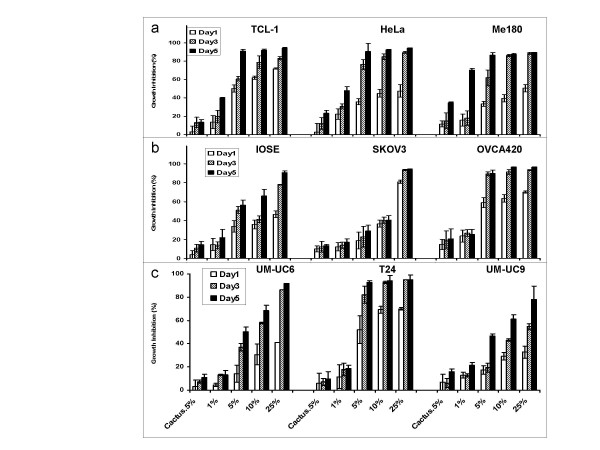
Effect of cactus pear extracts on growth of human cervical, ovarian, and bladder cancer cells in monolayer cultures. Cells were grown for 1, 3, or 5 days in the absence (control) or presence of 0.5, 1, 5, 10, or 25% of cactus pear extracts in (a.) immortalized cervical cells and cervical cancer cells; (b.) immortalized ovarian cells and ovarian cancer cells; and (c.) bladder cancer cells. Values are means ± SD of triplicate cultures. The percentage of growth inhibition (GI) was calculated using the equation: % GI = (1-Nt/Nc) × 100; where Nt and Nc represent the numbers of cells in treated and control cultures, respectively.

**Figure 3:**
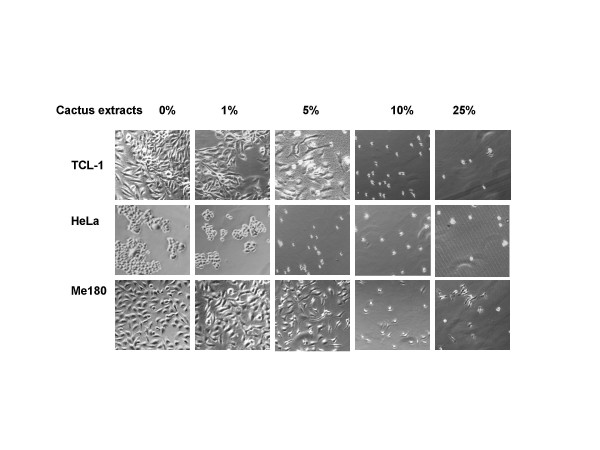
Effect of cactus pear extracts on the morphology of cervical cells. Immortalized cervical cells and cervical cancer cells were grown in the absence (control) or presence of different concentrations of cactus extracts. The photographs were taken on day 3 after the removal of medium containing floating cells.

**Figure 4:**
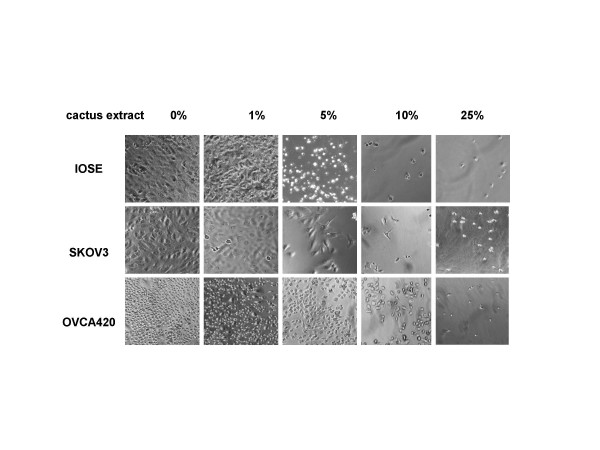
Effect of cactus pear extracts on the morphology of ovarian cells. Immortalized ovarian cells and ovarian cancer cells were grown in the absence (control) or presence of different concentrations of cactus extracts. The photographs were taken on day 3 after the removal of medium containing floating cells.

**Figure 5:**
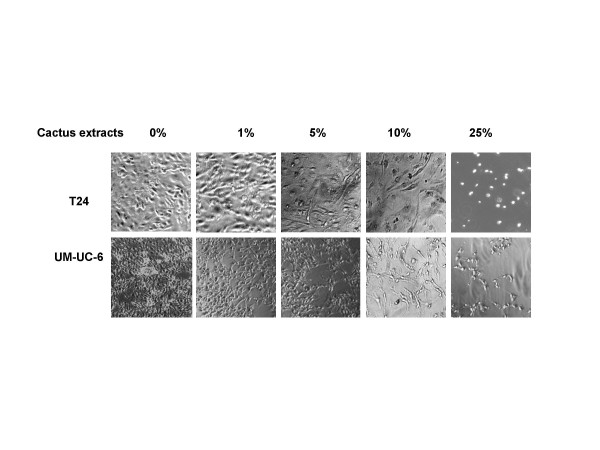
Effect of cactus pear extracts on the morphology of bladder cancer cells. Bladder cancer cells were grown in the absence (control) or presence of different concentrations of cactus extracts. The photographs were taken on day 3 after the removal of medium containing floating cells.

Cactus pear extracts were used at different concentrations (see Methods) to compare the inhibitory effect on a growth of 3 different types of human cancer cells in monolayer cultures. The sensitivity of cancer cells to cactus treatment differed among cell types. Cervical cancer cells were the most sensitive compared with ovarian and bladder cancer cells (Fig. 2a,b,c). One percent (1%) cactus pear solution inhibited 40–60% of immortalized cervical epithelium cells and cervical cancer cells (Fig. 2a). For ovarian cancer cells, 5% cactus pear solution was effective on growth inhibition in IOSE and OVCA420 cells, however, 10% solution was required to inhibit growth in SKOV3 cells (Fig. 2b). The concentration of cactus pear extracts effect on 50% of bladder cancer cell growth was greater than 1% (Fig. 2c). The effect of the cactus pear solution was dose-and time-dependent (Fig. 2). The IC50 (the concentration causing 50% cell death) in cervical and bladder cancer cells after 5-day treatment with cactus pear solution was less than 2 percent. For cervical cells, the IC50 for TCL-1 was 1.5%; HeLa was 1.8%; and ME180 was 0.8%. For bladder cancer cells, IC50 was 0.9% and 1.3% for UM-UC-6 and T24 cells, respectively. However, the IC50 for ovarian cells was varied, IC50 for IOSE, OVCA420, and SKOV3 cells were 2%, 0.8%, and 8%, respectively. Morphological changes were induced by cactus pear extracts 3 days after treatment and were in concordance with the agent's effect on cell growth of cervical cells (Fig. 3), ovarian cells (Fig. 4), and bladder cancer cells (Fig. 5).

### Apoptosis induction by cactus extract in different cancer cells

**Figure 6:**
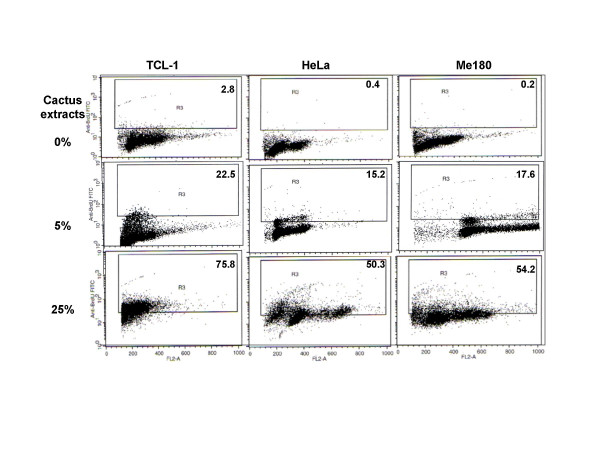
Apoptosis induction analyzed by TUNEL assay in cervical cells. Cells were treated with 5% and 25% cactus pear solution for 2 days. Cells were harvested and incubated with TdT in the presence of biotin-labeled BrdU and analyzed by flow cytometry. The percentage of apoptotic cells is represented by dark dots (fluorescence of individual cells) above the line in R3 region (R3 is the computer software analysis apoptosis program).

**Figure 7:**
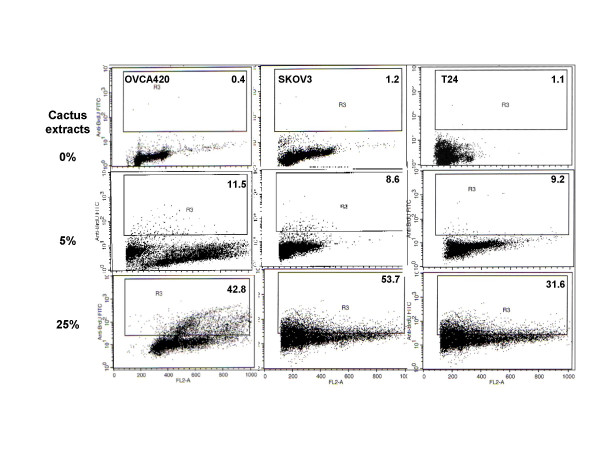
Apoptosis induction analyzed by TUNEL assay in ovarian and bladder cancer cells. TUNEL analysis results showed apoptosis induction by cactus extract in ovarian cancer cells (left and mid-panel) and bladder cancer cells (right panel).

Cactus pear solution induced apoptosis in all three cancer cell lines tested by TUNEL analysis (Fig. 6 and 7). In cancer cell lines, the strongest effect of apoptosis induction was found in cervical cells. The apoptosis cell population increased by more than 50% at the concentration of 25% cactus extract compared with the untreated cells (Fig. 6). This was consistent with cell growth inhibitory effects (Fig. 2 and 6). The immortalized cervical epithelium cells were the most sensitive in which the apoptotic cells increased over 70% after treatment (Fig. 6). Apoptosis induction in ovarian and bladder cancer cells differed: in ovarian cancer cells, cactus extracts increased apoptosis induction from 40% to 50% in OVCA420 and SKOV3 cells (Fig. 7, left and mid-panel). In T24 bladder cancer cells, apoptosis was 30% (Fig. 7, right panel). Apoptosis induction was not significant at 5% concentration.

### Cell cycle and apoptosis analysis in cancer cells

**Figure 8:**
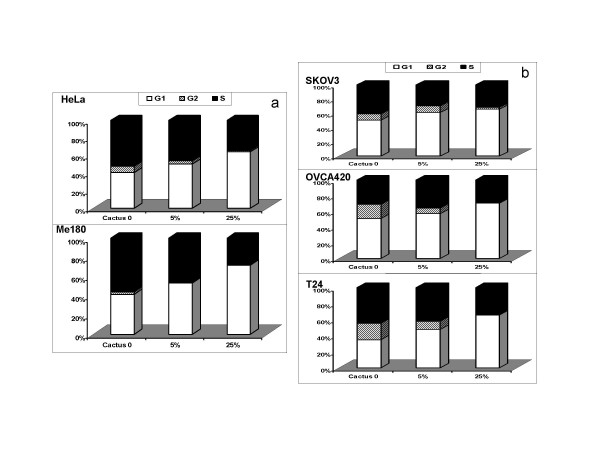
Cell cycle analysis. Cells were treated with 5 and 25% cactus pear extract for 2 days. Cells were stained with propidium iodide/RNase A solution for 30 min then analyzed by flow cytometry using a FACScan flow cytometer. (a.) cervical cancer cells HeLa and Me180; (b.) ovarian cancer cells SKOV3 and OVCA420 (upper), and bladder cancer cells T24 (bottom).

DNA content and cell cycle analysis were performed after treatment with 0, 5, and 25% concentrations of cactus pear solution. Results demonstrated that cactus pear extracts affected cell cycle in cancer cells starting at a 5% concentration (Fig. 8a, and 8b). In cervical cancer cells, cactus extracts increased cells in G1 and decreased those in the S phase (Fig. 8a). Treatment with higher concentrations of cactus pear extracts increased cells in G1 and decreased cells in G2 and in the S phase in ovarian and bladder cancer cells (Fig. 8b). The effect of cactus on cell cycle was dose-dependent.

### Cactus products inhibited tumor growth in a nude mice model

**Figure 9:**
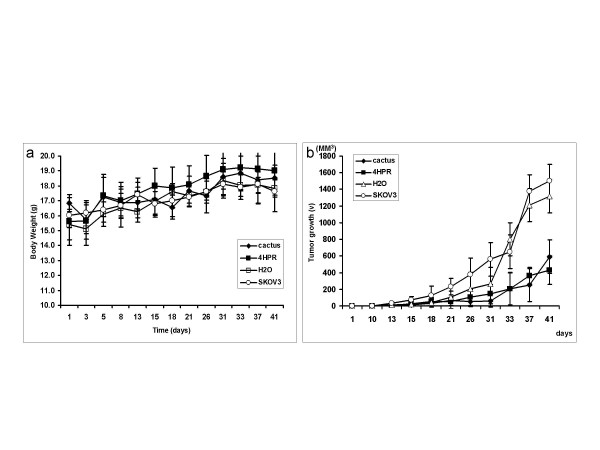
a. Animal body weight curve. The body weight was measured twice a week during the experiment. The picture represents the control animal labeled as H_2_O and treated-animal as SKOV3 only (SKOV3), SKOV3 + cactus pear (pear sol), and SKOV3 + 4HPR (4-HPR). b. Tumor growth curve. Tumor size in cactus pear and 4-HPR treatment groups, compared with control SKOV3 only and SKOV3 plus H_2_O, was significantly reduced (p < 0.05). The effect of cactus pear solution compared with 4-HPR on inhibiting tumor growth, both agents were able to inhibit SKOV3 inoculated tumor growth, the difference is not statistically significant (p > 0.05).

The treatment groups and the schedule of treatment are shown in Fig. 1. Animal body weight was measured twice a week for weight loss, as an indication of toxicity. Cactus pear extracts had no significant effect on weight loss (Fig. 9a) or animal behavior.

The cactus pear solution was able to inhibit tumor growth in nude mice compared with that in untreated animals or animals treated with H_2_O (Fig. 9b). The effect of cactus pear solution on inhibiting tumor growth indicated by tumor size was compared with 4-HPR, which is currently being used as a chemopreventive agent in ovarian, cervical and bladder cancer clinical trials [11-17] (Fig. 9b). We compared the control animal transplanted with SKOV3 cells only and SKOV3 + H_2_O to treatment group with either cactus pear extracts or 4-HPR. Cactus pear extracts and 4-HPR significantly reduced tumor size (p < 0.05). The inhibitory effect of 4-HPR was not significantly different from that of the cactus pear extract solution (p > 0.05).

### Immunohistochemistry staining for p53, annexin IV and VEGF expression

**Figure 10:**
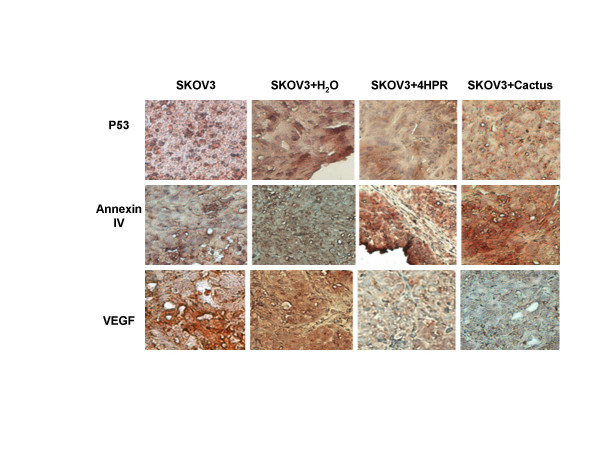
Representative immunohistochemistry patterns of p53, annexin IV and VEGF in animal tumor sections. p53 expression was stained as positive (+) in SKOV3 only and SKOV3 plus H_2_O groups, treatment of 4-HPR was slightly changes its expression and most of nuclei were stained negative (upper panel). Cactus extract treatment was found in some of nuclei stained negative (weak). Annexin IV expression was detected negatively (-) in SKOV3 only and SKOV3 plus H_2_O groups, treatment of both 4-HPR and cactus extracts were increased its expression (mid panel). VEGF expression was detected positively (+) in SKOV3 only and SKOV3 plus H_2_O groups, treatment of both 4-HPR and cactus extracts were decreased its expression (bottom panel).

The expression of p53, annexin IV, and VEGF were examined in animal tumor tissues. 4-HPR and cactus extracts treatment increased annexin IV and decreased VEGF expression; also cactus extracts had a stronger effect on suppression of VEGF expression (Fig. 10). Both 4-HPR and cactus extracts slightly changed p53 expression, where more negative nuclei were observed (Fig. 10).

## Discussion

Remarkable progress has been made over the past two decades in understanding the molecular and cellular mechanisms of pre-cancer and cancer progression [2]. Nonetheless, the development of effective and safe agents for prevention and treatment of cancer remains slow, inefficient, and costly [7], with little to offer the high-risk population for primary prevention and cancer survivors to prevent cancer recurrence. The key to effective chemoprevention is the identification of a chemopreventive agent(s) that can effectively inhibit cancer development without toxic side effects. In an Italian 4-HPR trial, retinoids showed the preventive effect on ovarian cancer only during the period while the drug was taken. After cessation of treatment, the incidence of ovarian cancer increased to the level that was observed in the untreated control group [10,11]. Therefore, chemopreventive agents may need to be used for a long period of time to be effective. As a result, identification of agents with little or no toxicity becomes important. We have shown that cactus pear extracts, a natural product, has anti-cancer activity, although the active component(s) have not been clearly identified. Since it has no toxic effects, cactus pear extracts can be easily used, for example, as dietary supplements [19-21] in normal and high risk populations.

It has been noted that Native Americans have a lower cancer rate when compared to white and African Americans [3]. Both cactus pear and nopale which contain multiple antioxidants, have been used as a dietary supplement for centuries by Native Americans. Our results show that the cactus pear inhibited growth of different cancer cells *in vitro *and *in vivo*. Cactus products inhibited cancer cell growth with concentrations as low as 5%; cell cycle was also affected at this concentration with an increase in G1 phase (Fig 2 and 8). However, apoptosis was observed at a higher concentration of 10% (data not shown) and 25% (Fig. 6 and 7).

We also compared cactus with the chemopreventive agent 4-HPR in nude mice. Both cactus and 4-HPR inhibited ovarian cancer growth. The anti-carcinogenic properties of natural and synthetic retinoids have been suggested to be due, in part, to the antioxidant effect [28-30], increased consumption of fruit and vegetables is associated with prevention of various human diseases, and the oxidative damage is an important etiologic risk factor for many diseases, including cancer and heart disease. Cactus pear extracts also contain multiple antioxidants that can reduce oxidative damage. The clinical trial on vitamin C and cactus pear demonstrated that supplements of vitamin C at a comparable dosage enhances overall antioxidant defense but does not significantly affect body oxidative stress [21,22]. Components of cactus pear extract, other than antioxidant vitamins, may play a role in anti-oxidant effects [21,22,31-33].

Carcinogenesis may be viewed as a process of progressive disorganization. This process is characterized by the accumulation of genotypic changes and corresponding tissue and cellular abnormalities including loss of proliferation and apoptosis controls. A dietary agent that can increase anti-proliferation pathways and change cell cycle in cancer cells without toxicity would be a potential agent for chemoprevention. Although the mechanism for cactus pear extract in cancer prevention is unclear, our current study shows that cactus pear does alter the expression of certain genes related to cell growth and apoptosis. Cactus pear extracts increased annexin IV and decreased VEGF expression in animal tumors. Annexin IV, a Ca2+-dependent membrane-binding protein, is expressed in many epithelial cancers [34]. Annexin IV played a pivotal role in the early phases of apoptosis [35], it was identified in initiation of apoptosis in human preneoplastic colonocytes [35], and its expression was regulated by quercetin [35]. Quercetin is one of the components of cactus pear extracts. Our results (unpublished data) and other reports [35,36] suggest quercetin might be one of the active compounds responsible for the anti-carcinogenetic and apoptosis-induction effects of cactus pear extracts. In our study, cactus pear extracts decreased VEGF expression, suggesting that cactus pear extracts might have inhibitory effects on angiogenesis, an important factor contributing to tumor growth and metastasis. We did not observe a significant effect on p53 expression caused either by 4-HPR or cactus pear extracts. Mutation of p53 is expected with the SKOV3 cell line, the tumor cells used in this animal model [37,38] but in this study, we observed minimal effect on p53 expression after treated with cactus extract and 4-HPR. However, since both wild-type and mutant p53 could contribute to induction of apoptosis, involvement of p53 pathway by 4-HPR or cactus pear extract cannot be ruled out by these results.

For developing food-derived agents, the NCI has advocated co-development of a single or purified extract of a few putative active compounds that are contained in food-derived agents [7]. The cactus pear extracts tested in this study could be such a candidate in cancer prevention for both normal and high-risk populations and prevention of recurrence in patients with previous cancers. This product holds promise for long-term use because of the safety of food-derived products and the fact that they are not perceived as a "chemical".

## Conclusion

Arizona prickly pear cactus effectively inhibited cell growth in several different immortalized and cancer cell cultures *in vitro *and suppressed tumor growth in a nude mouse of ovarian cancer model. The mechanism of anti-cancer effect of cactus pear extracts is not yet completely understood. Currently, we are investigating the expression of genes related to cell growth and apoptosis which may be altered by treatment with cactus products to elucidate possible pathways through which this natural product exerts its anti-cancer effects.
